# Novel regulatory role of neuropilin-1 in endothelial-to-mesenchymal transition and fibrosis in pancreatic ductal adenocarcinoma

**DOI:** 10.18632/oncotarget.11060

**Published:** 2016-08-11

**Authors:** Pratiek N. Matkar, Krishna Kumar Singh, Dmitriy Rudenko, Yu Jin Kim, Michael A. Kuliszewski, Gerald J. Prud'homme, David W. Hedley, Howard Leong-Poi

**Affiliations:** ^1^ Division of Cardiology, Keenan Research Centre for Biomedical Science, St. Michael's Hospital, Toronto, Canada; ^2^ Division of Vascular Surgery, Keenan Research Centre for Biomedical Science, St. Michael's Hospital, Toronto, Canada; ^3^ Division of Cardiac Surgery, Keenan Research Centre for Biomedical Science, St. Michael's Hospital, Toronto, Canada; ^4^ Division of Laboratory Medicine, Keenan Research Centre for Biomedical Science, St. Michael's Hospital, Toronto, Canada; ^5^ Division of Medical Oncology and Hematology, Ontario Cancer Institute, Campbell Family Cancer Research Institute, Princess Margaret Cancer Centre, Toronto, Canada; ^6^ Institute of Medical Science, University of Toronto, Toronto, Canada

**Keywords:** endothelial cell, neuropilin-1, endothelial-to-mesenchymal transition, fibrosis, pancreatic cancer

## Abstract

Pancreatic ductal adenocarcinoma (PDAC) is characterized by an intense fibrotic reaction termed tumor desmoplasia, which is in part responsible for its aggressiveness. Endothelial cells have been shown to display cellular plasticity in the form of endothelial-to-mesenchymal transition (EndMT) that serves as an important source of fibroblasts in pathological disorders, including cancer. Angiogenic co-receptor, neuropilin-1 (NRP-1) actively binds TGFβ1, the primary mediator of EndMT and is involved in oncogenic processes like epithelial-to-mesenchymal transition (EMT). NRP-1 and TGFβ1 signaling have been shown to be aberrantly up-regulated in PDAC. We report herein a positive correlation between NRP-1 levels, EndMT and fibrosis in human PDAC xenografts. Loss of NRP-1 in HUVECs limited TGFβ1-induced EndMT as demonstrated by gain of endothelial and loss of mesenchymal markers, while maintaining endothelial cell architecture. Knockdown of NRP-1 down-regulated TGFβ canonical signaling (pSMAD2) and associated pro-fibrotic genes. Overexpression of NRP-1 exacerbated TGFβ1-induced EndMT and up-regulated TGFβ signaling and expression of pro-fibrotic genes. *In vivo*, loss of NRP-1 attenuated tumor perfusion and size, accompanied by reduction in EndMT and fibrosis. This study defines a previously unrecognized role of NRP-1 in regulating TGFβ1-induced EndMT and fibrosis, and advocates NRP-1 as a therapeutic target to reduce tumor fibrosis and PDAC progression.

## INTRODUCTION

Despite improvements in our understanding of cancer biology and advances in cancer therapies, malignant tumors remain a leading cause of morbidity and mortality [1–3]. Pancreatic adenocarcinoma (PDAC) is a leading cause of mortality worldwide, with the lowest five-year survival rate [1, 4–6]. As a consequence, the advancement of novel therapeutic strategies that specifically target the underlying biological processes involved in pancreatic cancer growth and metastases remains important [7]. Given the critical role of angiogenesis in cancer biology, anti-angiogenic therapies have been in the forefront of cancer therapeutics for decades [8–11]. Less vascularized tumors like PDAC, however remain largely incurable despite the significant advances in anti-angiogenesis research [12, 13]. Morphologically, PDAC is composed of epithelial cells with variable degrees of ductal differentiation, surrounded by an intense fibrotic reaction called tumor desmoplasia, often referred to as tumor stroma/fibrosis [14–18]. Cancer-associated fibroblasts (CAFs) along with other stromal cells constitute the majority of the PDAC tumor [19, 20]. Recent evidence has demonstrated that CAFs contribute to several cancer initiating and promoting events by altering the tumor microenvironment through the release of oncogenic and angiogenic factors [20–22]. Furthermore, the characteristically dense desmoplastic reaction found in PDAC, including the stromal cells, extracellular matrix components and high interstitial pressure within the PDAC tumors can impair drug delivery and collectively contribute towards chemoresistance [13, 23–25]. Recently approved therapies targeting desmoplasia/fibrosis [26–28] opens new avenues for discovering novel strategies to suppress desmoplasia.

Although the origin of fibroblasts is complex and dependent on several factors, the importance of endothelial-to-mesenchymal transition (EndMT) as an important source of activated fibroblasts is an emerging concept [29–31]. Besides the crucial function carried out as a part of the endothelium, endothelial cells have displayed the ability of acquiring an extreme form of cell plasticity, whereby they acquire a mesenchymal cell phenotype. Primarily, EndMT is characterized by the acquisition of mesenchymal cell surface markers such as N-cadherin, αSMA and types I/III collagen with the corresponding loss of endothelial cell surface markers like VE-cadherin, CD31, etc. [29, 31]. While tightly regulated, EndMT is critical in the development of the primitive heart [32] and wound healing [33], and maladaptive EndMT has been linked to a variety of fibrotic pathologies including cancer [31, 34]. Zeisberg *et al*. demonstrated convincing genetic evidence for EndMT-derived CAFs in the tumor microenvironment, whereby up to 40% of CAFs were derived via EndMT [35]. Mechanistically, several studies have established the involvement of TGFβ signaling in modulating EndMT [36–39]. However, mechanistic work involving EndMT in the context of diseased states like cancer remains obscure and warrants further investigation.

Initially implicated in the development of the nervous system [40], growing evidence suggests a significant role for neuropilin-1 (NRP-1) in cancer where it appears to be involved in angiogenesis and other aspects of tumor progression [41–48]. NRP-1 has been identified as a co-receptor for multiple growth factors, including VEGF-A, FGF, HGF and others [44, 49–52], and is expressed on both endothelial and tumor cells [53, 54]. Our group has previously demonstrated that NRP-1 is also a co-receptor for TGFβ1, where it could enhance responses to both the active and latent forms of this cytokine [51]. Emerging data support TGFβ1 as a major modulator of tumor progression through regulation of endothelial cell proliferation and migration, extracellular matrix metabolism, epithelial-to-mesenchymal transition (EMT) and metastasis [55–59]. Furthermore, PDAC is characterized by aberrant expression of NRP-1 [45, 60, 61], often correlated with tumor progression and poor patient prognosis [62]. Interestingly, recent evidences have implicated a novel regulatory role of NRP-1 in epithelial-to-mesenchymal transition (EMT); an evolutionary conserved developmental process, which is evoked during tumor invasion and metastasis of several cancers [63, 64]. Similarly, recent studies suggest that endothelial-to-mesenchymal transition (EndMT), which is originally observed during heart development, has a major role in pathological settings of cancer and fibrosis [21–25, 31]. Transforming growth factor β (TGFβ) plays pivotal roles in both EMT and EndMT [65, 66]. Finally, the precise function of NRP-1 in TGFβ-mediated EndMT becomes highly relevant, given the importance of the pro-fibrotic cytokine in tumor progression [22, 58, 67, 68], and the involvement of NRP-1 in pancreatic cancer and EMT.

In the current study, we hypothesized that NRP-1 is crucial in regulating TGFβ1-induced EndMT and associated fibrosis in PDAC. To test our hypothesis, we utilized translational loss of function and over-expression study approaches. Firstly, we demonstrate a robust positive correlation between NRP-1 expression, EndMT and associated fibrosis in human PDAC xenografts. Using loss of function and overexpression studies *in vitro*, we demonstrate a novel regulatory role of NRP-1 in TGFβ1-induced EndMT and fibrosis. Knockdown of NRP-1 limited while NRP-1 overexpression exacerbated TGFβ1-induced EndMT respectively. Overall, these data reveal a previously undetermined role of NRP-1 in regulating TGFβ1-induced EndMT as a potential therapeutic target to limit EndMT-associated fibrosis in PDAC.

## RESULTS

### Human PDAC tissue shows positive correlation between NRP-1 expression, EndMT and fibrosis

**Figure 1:**
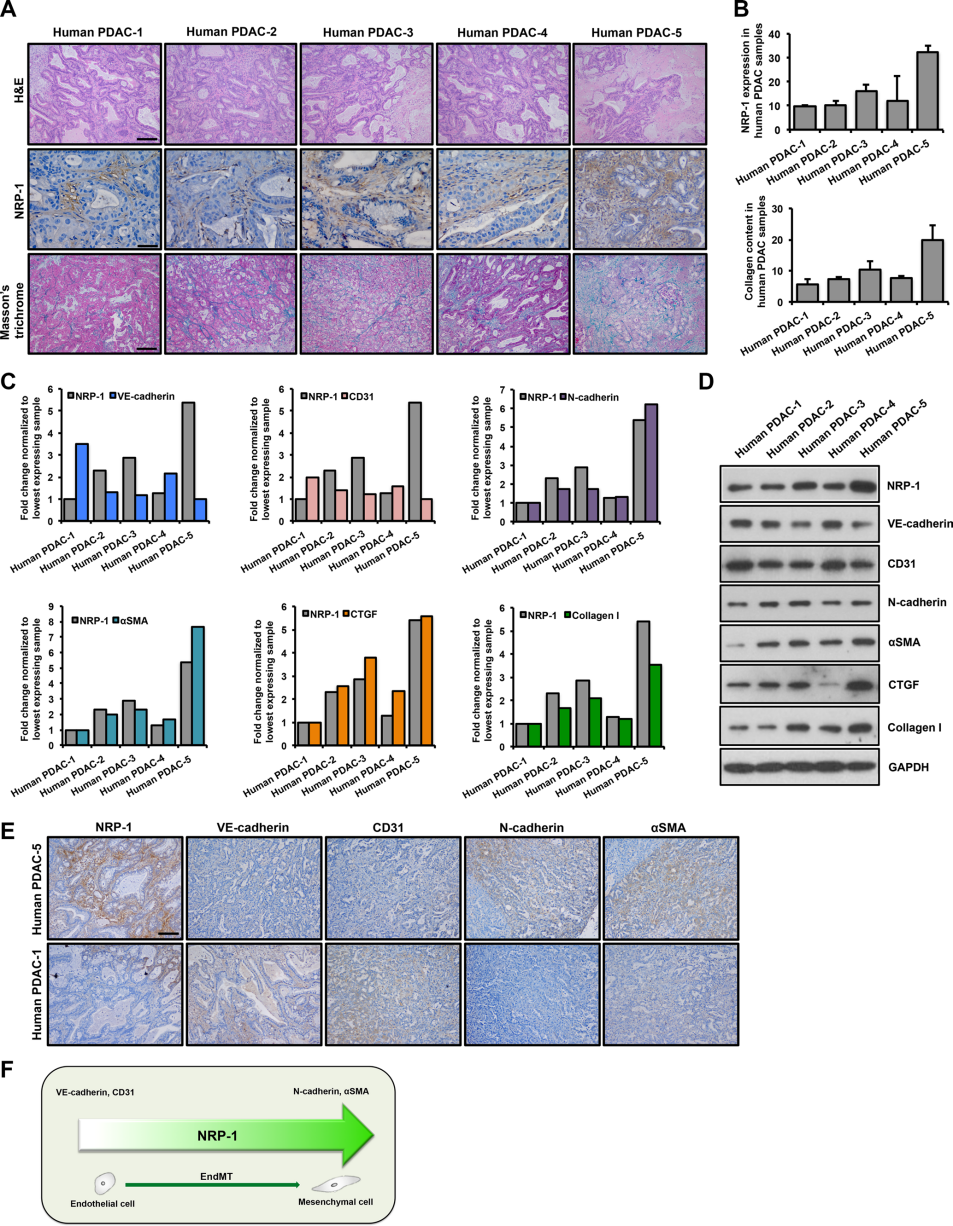
NRP-1 levels positively correlate with EndMT and fibrosis markers in human PDAC xenografts Five human PDAC xenografts were obtained for this study. (**A**) Tumor tissues were stained with H&E staining to recognize tissue type and the morphology; for NRP-1 levels (indicated by brown color) by immunohistochemistry and for extent of fibrosis by Masson's trichrome staining (indicated by blue color). Representative images reveal varying levels of tissue differentiation (Scale bar = 100 μm), NRP-1 expression (Scale bar = 50 μm) and extent of fibrosis in the xenografts (Scale bar = 100 μm). (**B**) Quantification of NRP-1 levels and collagen content using the ImageJ software (expressed in AU, arbitrary units). NRP-1 levels positively correlate with EndMT markers and pro-fibrotic genes at the transcript (**C**) and protein level (**D**) as determined by qPCR and western blotting respectively. Correlation was determined by Spearman's Rho test (positive correlation: 0.8 < R ≤ 1, R = correlation coefficient). For C, data has been normalized to tissue sample having lowest expression of the respective genes that are plotted. (**E**) Representative immunohistochemistry images showing correlation between NRP-1 level and EndMT markers in tumor tissues having the lowest and highest NRP-1 expression as determined previously (Scale bar = 100 μm). (**F**) Summary of NRP-1-EndMT correlation.

H&E staining of human tumor tissues displayed a typical ductal morphology of PDAC (Figure 1A). Further characterization of the xenografts is summarized in Tables 1 and 2. NRP-1 was aberrantly expressed in all the tissue samples at varying levels as assessed by immunohistochemistry studies (Figure 1A). Furthermore, collagen content as detected by Masson's trichrome staining, demonstrated a significant positive correlation with NRP-1 expression (Figure 1A, 1B). We observed a consistent increase in the expression of EndMT markers and pro-fibrotic markers with increasing NRP-1 expression at the transcript (Figure 1C) and protein level (Figure 1D, 1E). Contrarily, low levels of EndMT and pro-fibrotic markers were associated with reduced NRP-1 expression at the transcript and protein level (Figure 1C, 1D, 1E). Overall, we observed a robust positive correlation between NRP-1 expression, EndMT markers and pro-fibrotic genes in human PDAC tissue (Figure 1F), suggesting a previously undetermined role of NRP-1 in regulating EndMT and associated fibrosis in pancreatic tumors.

**Table 1:** Partial characterization of pancreatic cancer xenografts established from patients

Human PDAC Xenograft	Sex	Tumor Grade	Clinical Stage^1^	Pathology Stage^1^	Undergone Surgery	Relapse	Survival (In days)
1 (OCIP23)	M	G3	T3NxM0	T3N1b	Yes	Distant	249
2 (OCIP28)	F	G1	T4N0M0	T3N0	Yes		2047*
3 (OCIP217)	F	G3	T4N0M1	T2N0	Yes	Distant	777
4 (OCIP167)	M	G2	T2NxM0	T3N1b	Yes	Distant	1150
5 (OCIP232)	M	G2	TxNxM1	T3N1b	Yes	Distant	681

Abbreviations: M-male; F-female. Data has been adapted from Lohse *et al*. [85].

1 TNM classification of tumors of the exocrine pancreas: T2, tumor limited to the pancreas, more than 2 cm in greatest dimension; T3, tumor extends directly into any of the following: duodenum, bile duct, peripancreatic tissues; T4, tumor extends directly into any of the following: stomach, spleen, colon, adjacent large vessels; N0, no regional lymph node metastasis; N1a, metastasis in a single regional lymph node; N1b, metastasis in multiple regional lymph nodes.

* Patient alive when the study was published.

**Table 2:** Summary of response to treatment

Human PDAC Xenograft	Radio-therapy	Chemotherapy	Response to Treatment
1 (OCIP23)	No	Gemcitabine*	Progression of disease
2 (OCIP28)	Yes	Gemcitabine-Cisplatin#	Partial therapeutic response
		Capecitabine*	No indication of disease
3 (OCIP217)	Yes	Gemcitabine-Cisplatin#	Partial therapeutic response
		Gemcitabine-Cisplatin+	Stable disease
		Carboplatin–paclitaxel	Progression of disease
4 (OCIP167)	No	Gemcitabine*	No indication of disease
		5FU–irinotecan oxaliplatin+	Stable disease
5 (OCIP232)	No	Gemcitabine*	No indication of disease
		5FU–irinotecan–oxaliplatin–leucovorin+	Initial therapeutic response but progressed after 6 months
		Veliparib+	Progression of disease

Abbreviations: 5FU- 5-fluorouracil. Data has been adapted from Lohse *et al*. [85].

* Adjuvant,

# Neoadjuvant,

+ Palliative.

### NRP-1 siRNA leads to successful knockdown at the transcript, protein and functional levels in HUVECs

**Figure 2:**
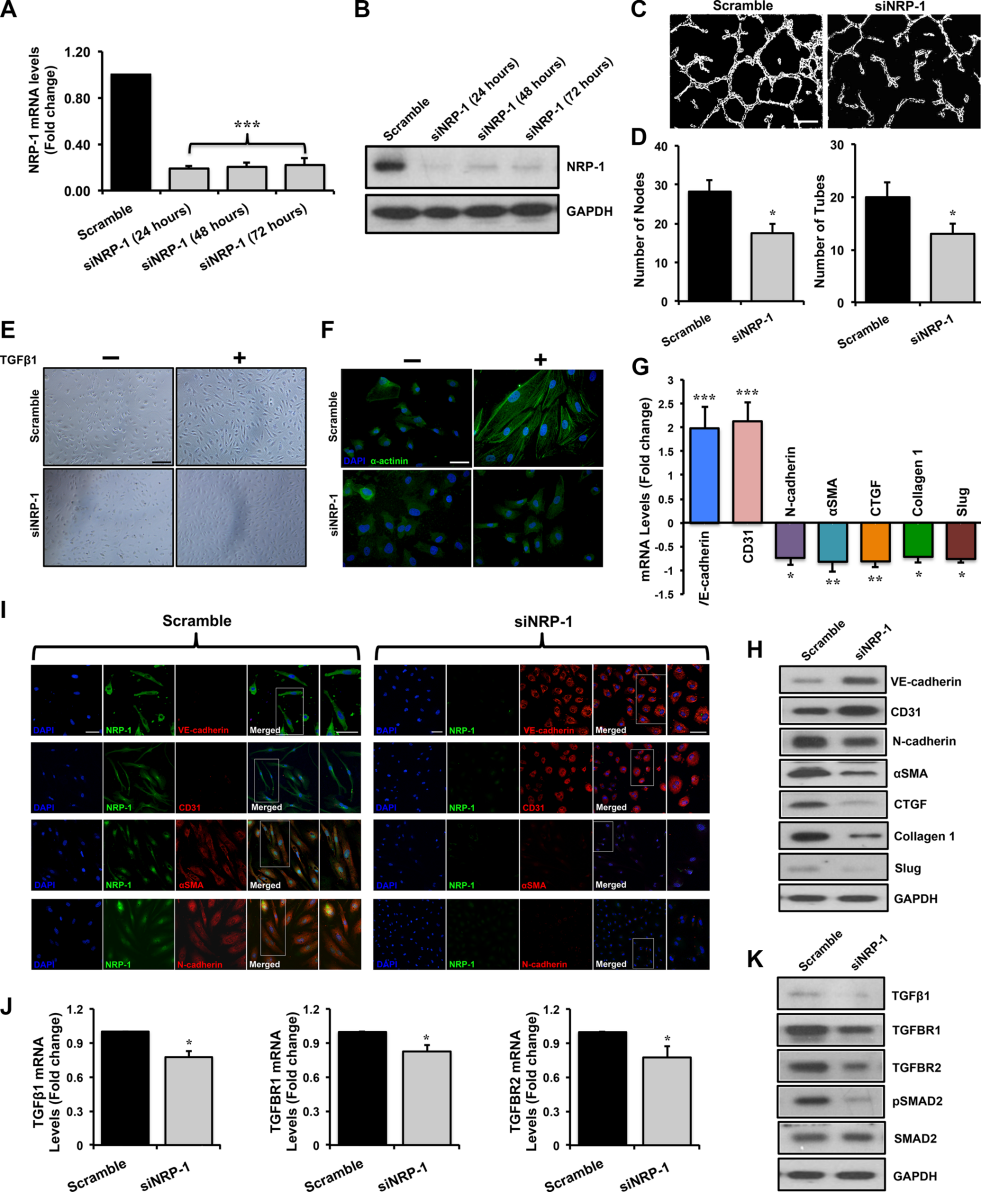
Loss of NRP-1 inhibits TGFβ1- induced EndMT in HUVECs. HUVECs were cultured and transfected with 5 nM of either siNRP-1 or scramble control Total RNA and protein was extracted from the transfected HUVECs at 24, 48 and 72 h post-transfection. (**A**) qPCR data demonstrate successful silencing of NRP-1 (~85% reduction) in siNRP-1 transfected HUVECs after 24 h. All qPCR data are presented as fold change to the scrambled control. (**B**) NRP-1 silencing was confirmed at protein level by immunoblotting at 24, 48 and 72 h after transfection. GAPDH was used as a loading control. (**C**) Representative images from capillary-like tube formation assay demonstrating reduced number of tubes in siNRP-1 compared to scramble control at 6 h. (**D**) Tube formation ability of HUVECs on Matrigel^TM^ was quantified as number of nodes and number of tubes, showing that siNRP-1 caused a significant anti-angiogenic effect as compared to scramble control. *p < 0.05 and ***p < 0.001 vs. scramble control. *n* = 5 in triplicate. (**E**) Confluent monolayer of HUVECs exhibits typical cobblestone morphology in scramble control under light microscope (20X, scale = 50 μm). TGFβ1 stimulation (10 ng/ml) caused marked morphological changes with the cells becoming enlarged and spindle shaped in scramble control transfected cells. These morphological changes were inhibited to some extent in siNRP-1 transfected TGFβ1-stimulated HUVECs. (**F**) Fluorescent microscopy images (scale = 10 μm) stained with alpha-actinin (α-actinin; green color), demonstrating cytoskeletal protein re-organization in scramble control transfected TGFβ1-stimulated HUVECs. Nuclei were stained with DAPI (blue color). HUVECs were transfected with scramble control and siNRP-1, and total RNA and protein was extracted at 24 h and 48 h, respectively. qPCR analysis demonstrates significant changes in the EndMT markers at transcript level (**G**) and protein level (**H**) by immunoblotting. These data were further confirmed by immunofluorescence for NRP-1 and EndMT markers (**I**) (scale = 20 μm; scale for magnified image = 10 μm) 48 h post-transfection. Nuclei were stained by DAPI (blue). (**J**) qPCR data analysis demonstrates significant down-regulation of TGFβ1, TGFBR1 and TGFBR2 and TGFβ1-responsive genes; Slug, Collagen I and CTGF at transcript level and (**K**) TGFβ1, TGFBR1, TGFBR2, pSMAD2, SMAD2 and (H) pro-fibrotic genes CTGF, Collagen I and Slug at protein level by immunoblotting upon NRP-1 silencing. *p < 0.05, **p < 0.01, ***p < 0.0001 vs. scramble control. *n* = 3–4 in triplicate.

We performed siRNA-mediated knockdown of *NRP-1* in HUVECs. *In vitro* knockdown studies were performed in three groups: 1. non-transfected control, 2. transfected scramble control and 3. transfected siNRP-1. Data from second and third groups are shown in the manuscript since data from the non-transfected control and scramble control transfected HUVECs were similar (data not shown). NRP-1 knockdown was confirmed by a significant reduction in NRP-1 expression by qPCR and immunoblotting analysis at different time points (Figure 2A, 2B). NRP-1 is primarily involved in angiogenesis through its co-receptor function with VEGFR-2 by binding to VEGF-A [44]. We therefore performed the capillary-like tube formation assay in HUVECs to assess NRP-1 knockdown at functional level and observed that siNRP-1 demonstrated a significant reduction of number of nodes and tubes as compared to scramble control (Figure 2C, 2D).

### Loss of NRP-1 inhibits TGFβ1-induced phenotypic switching typical of endothelial-to-mesenchymal transition in HUVECs

Light microscopy studies showed that TGFβ1-stimulated scramble-transfected HUVECs demonstrated a distinctive morphological change from typical ‘cobblestone-like endothelial cell morphology’ to an enlarged spindle shaped manifestation, characteristic of ‘mesenchymal-like cell morphology’ (Figure 2E). Additionally, we observed that these morphological changes were associated with cytoskeletal protein re-organization. In accordance, we observed an increase in α-actinin expression and a representative mesenchymal cell-like cytoskeletal protein re-organization (Figure 2F). Interestingly, these distinctive morphological and ultra-structural protein re-organization changes were inhibited upon loss of NRP-1 (Figure 2E, 2F). Phenotypic EndMT characteristics are accompanied by corresponding changes in the expression of cell surface markers [24, 26]. In keeping with characteristics of reversal of EndMT, upon TGFβ1 stimulation, loss of NRP-1 significantly induced the expression of endothelial markers; VE-cadherin and CD31, and inhibited the expression of mesenchymal markers; αSMA, N-cadherin and transcription factor Slug as assessed by qPCR (Figure 2G), immunoblotting (Figure 2H) and immunofluorescence studies (Figure 2I) when compared to scramble control transfected cells.

Mechanistically, TGFβ signaling is crucial in regulating EndMT [36–38]. Knockdown of NRP-1 in TGFβ1-stimulated HUVECs resulted in significantly reduced expression of TGFβ1 ligand at the transcript (Figure 2J) and protein (Figure 2K) level when compared to the scramble control. The TGFβ1 isoform uses the same set of receptors as other isoforms consisting of TGFBR1 and TGFBR2 [47]. TGFβ1 binds to TGFBR2 initially, which further bind to TGFBR1 to form a complex with serine/threonine kinase activity, where TGFBR2 phosphorylates TGFBR1. This complex is further capable of phosphorylating downstream molecules belonging to the SMAD family of proteins that possess the transcription regulatory activity upon translocation to the nucleus. Knockdown of NRP-1 down-regulated TGFBR1 and TGFBR2 at transcript and protein levels as assessed by qPCR and immunoblotting, respectively, in TGFβ1-stimulated HUVECs (Figure 2J, 2K). Loss of NRP-1 was further accompanied by notably reduced SMAD2 phosphorylation as demonstrated by immunoblotting (Figure 2K, Supplementary Figure 1A, 1B). Upon investigating the expression of TGFβ1-dependent pro-fibrotic genes, like CTGF and Collagen 1, we observed that loss of NRP-1 in TGFβ1-stimulated HUVECs significantly decreased their expression at the protein level as detected by immunoblotting (Figure 2H). As anticipated, reduction in SMAD2 phosphorylation and expression of pro-fibrotic genes was not observed in the scramble control HUVECs group (Figure 2H, 2K). Intriguingly, in the absence of TGFβ1 stimulation, loss of NRP-1 significantly increased the expression of endothelial markers; VE-cadherin and CD31, however, the decrease in expression of mesenchymal markers; N-cadherin and αSMA, although moderately reduced, was not significant as assessed by qPCR (Supplementary Figure 1F).

### Lentivirus-mediated NRP-1 transduction in HUVECs leads to successful overexpression at the transcript, protein and functional levels

**Figure 3:**
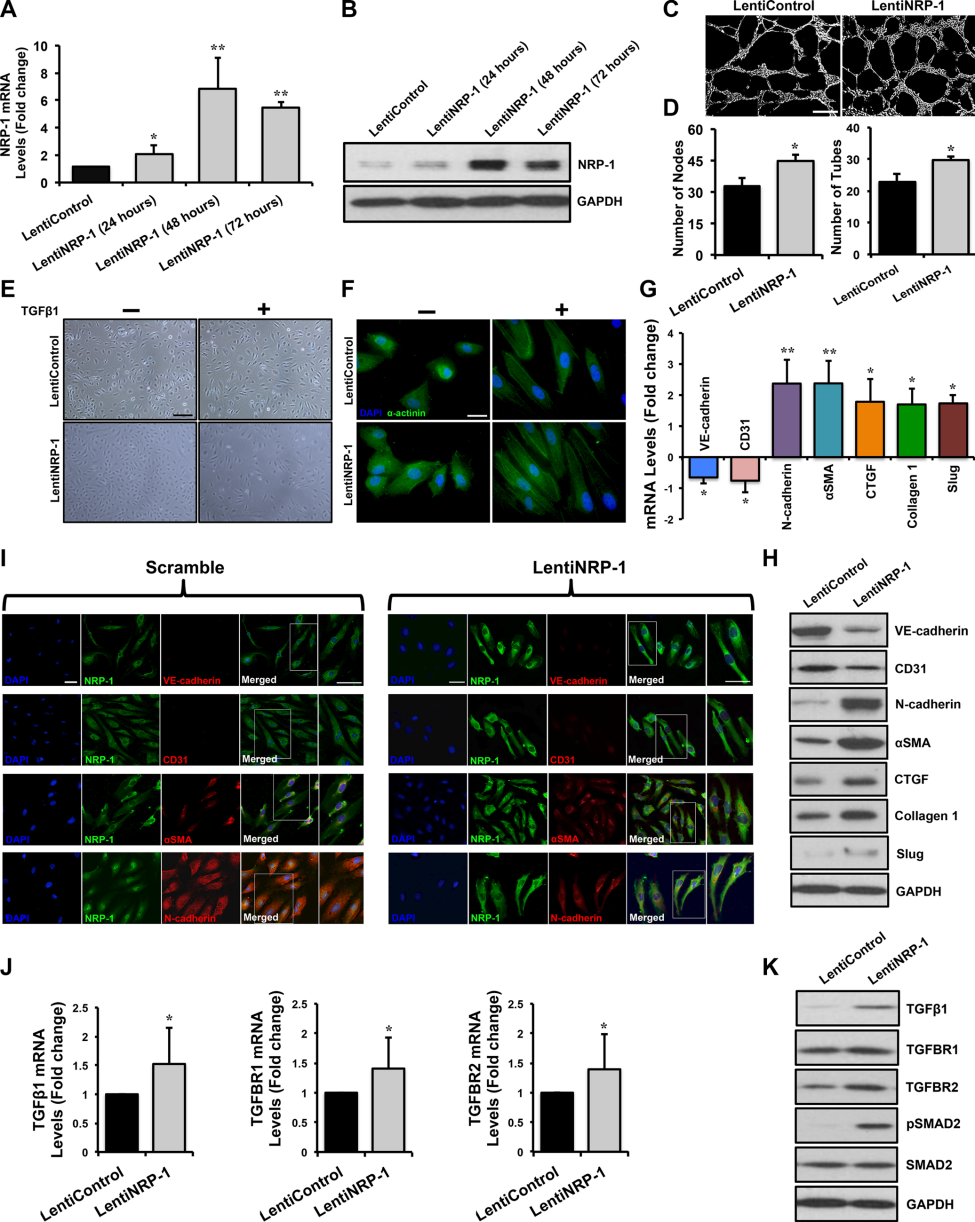
Lentivirus-mediated NRP-1 overexpression exacerbates TGFβ1-induced EndMT in HUVECs HUVECs were cultured and transduced with either lentiNRP-1 or blank/empty control lentivirus (lentiControl). Total RNA and protein was extracted from the transduced HUVECs at 24, 48 and 72 h post-transduction. (**A**) qPCR data demonstrate successful overexpression of NRP-1 in lentiNRP-1 transduced HUVECs after 48 h. All qPCR data are presented as fold change to the scrambled control. (**B**) NRP-1 overexpression was confirmed at protein level by immunoblotting. (**C**) Representative images from capillary-like tube formation assay demonstrating increased number of tubes in lentiNRP-1 compared to lentiControl at 6 h time point. (**D**) Tube formation ability of HUVECs on Matrigel^TM^ was quantified as number of nodes and number of tubes and showed that lentiNRP-1 caused a significant pro-angiogenic effect as compared to lentiControl. *p < 0.05 and **p < 0.01 vs. lentiControl. *n* = 5. (**E**) Confluent monolayer of HUVECs exhibits typical cobblestone morphology in scramble control under light microscope (scale = 50 μm). TGFβ1 stimulation caused marked morphological changes with the cells becoming enlarged and spindle shaped in cells transduced with lentiControl. Similar morphological changes were observed in lentiNRP-1 transduced HUVECs upon TGFβ1 stimulation. (**F**) Fluorescent microscopic images (scale = 10 μm) stained with alpha-actinin (α-actinin; green color), demonstrating cytoskeletal protein re-organization in lentiNRP-1 transduced HUVECs. Nuclei were stained with DAPI (blue color). HUVECs were transduced with lentiControl and lentiNRP-1, and total RNA and protein was extracted at 48 h. (**G**) qPCR analysis demonstrates significant changes in the EndMT markers at transcript level and protein level by immunoblotting (**H**). (**I**) These data were further confirmed by immunofluorescence for NRP-1 and EndMT markers (Scramble: scale = 20 μm, scale for magnified image = 10 μm; LentiNRP-1: scale = 10 μm, scale for magnified image = 10 μm) 48 h post-transduction in the photomicrographs. Nuclei were stained by DAPI (blue). (**J**) qPCR data analysis demonstrates significant down-regulation of TGFβ1, TGFBR1, TGFBR2 and (G) TGFβ1-responsive genes Slug, Collagen 1 and CTGF at transcript level and (**K**) TGFβ1, TGFBR1, TGFBR2, pSMAD2, SMAD2 and (H) pro-fibrotic genes Slug, Collagen I and CTGF at protein level by immunoblotting upon NRP-1 overexpression. Nuclei were stained by DAPI (blue) *p < 0.05, **p < 0.01 and ***p < 0.001 vs. lentiControl. *n* = 3–4 in triplicate.

We performed lentiviral-mediated overexpression of *NRP-1* in HUVECs. *In vitro* overexpression studies were performed in three groups; 1. non-transduced control, 2. transduced lentiControl and 3. transduced lentiNRP-1. Data from empty control (LentiControl) and lentiNRP-1 are shown in the manuscript since data from the non-transduced control and empty control transduced HUVECs were indistinguishable (data not shown). NRP-1 overexpression was confirmed by a striking increase in NRP-1 expression by qPCR (Figure 3A) and immunoblotting (Figure 3B) analysis at different time points. The capillary-like tube formation assay exhibited that lentiNRP-1 demonstrated a significant increment in number of nodes and tubes as compared to lentiControl owing to the co-receptor function of NRP-1 during VEGF-induced angiogenesis (Figure 3C, 3D).

### Overexpression of NRP-1 in TGFβ1-stimlated HUVECs induces distinct morphological and molecular changes consistent with EndMT

Light microscopy studies showed that overexpression of NRP-1 in TGFβ1-stimlated HUVECs lead to a distinctive morphological change, in accordance with phenotypic switching associated with EndMT (Figure 3E). Furthermore, we observed an increase in α-actinin expression and a mesenchymal cell-like cytoskeletal protein re-organization upon NRP-1 overexpression as compared to lentiControl (Figure 3F). In agreement with TGFβ1-induced EndMT characteristics, NRP-1 overexpression significantly down-regulated the expression of endothelial markers; VE-cadherin and CD31, and induced the expression of the mesenchymal markers; N-cadherin, αSMA and Slug as assessed by qPCR (Figure 3G), immunoblotting (Figure 3H) and immunofluorescence (Figure 3J). Interestingly, NRP-1 overexpression in the absence of TGFβ1 stimulation resulted in an increase in mesenchymal markers' expression but no significant decrease in the expression of all the assessed endothelial cell markers (Supplementary Figure 1G).

Examining at the mechanistic level, we observed that overexpression of NRP-1 in TGFβ1-stimulated HUVECs resulted in significantly elevated expression of TGFβ1 at the transcript and protein level when compared to lentiControl (Figure 3J, 3K). Additionally, overexpression of NRP-1 up-regulated TGFBR1 and TGFBR2 at transcript and protein level as assessed by qPCR (Figure 3J) and immunoblotting (Figure 3K) respectively, and led to increased SMAD2 phosphorylation as demonstrated by immunoblotting (Figure 3K). Overexpression of NRP-1 along with TGFβ1-stimulation significantly augmented the expression of TGFβ1-dependent pro-fibrotic genes, like CTGF and Collagen 1 at transcript and protein level as compared to lentiControl (Figure 3G, 3H). Thus our NRP-1 overexpression study was antagonistic with our loss of NRP-1 function study, and in accordance with our hypothesis. This confirms the novel *in vitro* regulatory role of NRP-1 in TGFβ1-induced EndMT.

### Loss of NRP-1 inhibits EndMT and fibrosis *in vivo* and results in reduced tumor growth

**Figure 4:**
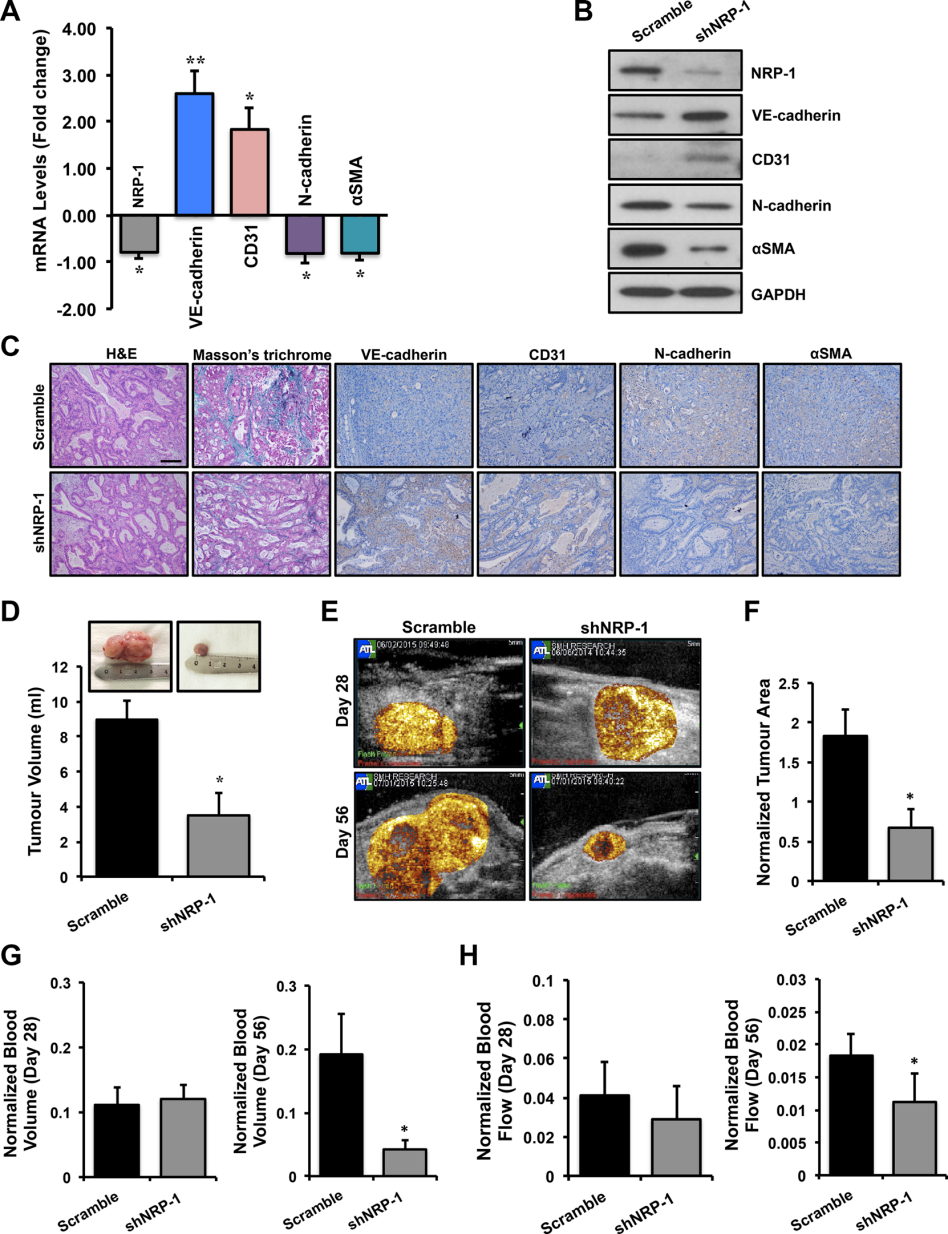
Loss of NRP-1 inhibits EndMT and fibrosis *in vivo* and results in reduced tumor growth Total RNA and protein was extracted from the tumor tissues. (**A**) qPCR data and (**B**) immunoblotting demonstrate successful silencing of NRP-1 in shNRP-1 minicircle delivered animals. (A) qPCR analysis demonstrates significant changes in the EndMT markers at transcript level and protein level (B) by immunoblotting. (**C**) These data were further confirmed by immunohistochemistry for NRP-1 and EndMT markers (scale = 100 μm; indicated by brown color). The tumor tissues were also stained with H&E to recognize tissue type and the morphology, and extent of fibrosis by Masson's trichrome staining (indicated by blue color). (C) Representative images reveal ductal morphology, reduced NRP-1 levels and reduced extent of fibrosis in in shNRP-1 minicircle delivered animals. (**D**) Tumor volumes (ml) as measured by volumetric assessment, with smallest tumor volumes seen in the shNRP-1 minicircle group as compared to scramble group. (**E**) Representative contrast-enhanced ultrasound perfusion images of orthotopic pancreatic tumors on day 28 (before gene delivery) and day 56 (28 days after gene delivery). Images are color coded, with bright yellow/orange signifying the highest acoustic signal and greatest perfusion. Tumor area (**F**) and perfusion (**G, H**) is greater and more complete throughout the tumor in scramble minicircle group, while reduced perfusion seen in tumors treated with shNRP-1 minicircle. (G,H) At day 28 there were no significant differences in microvascular blood volume (MBV) and microvascular blood flow (MBF) between groups. Normalized tumor blood volume (G) and blood flow (H) at day 56. At day 56, tumor blood volume and flow was significantly reduced in shNRP-1 minicircle group compared with scramble control group. *p < 0.05, **p < 0.01 vs. scramble. *n* = 4 for each group. Tumor area and volume data are expressed as mean ± SEM.

Prior to *in vivo* studies, the knockdown efficiency of the shNRP-1 minicircle was tested *in vitro* in BxPC-3 cells. NRP-1 knockdown was confirmed by a significant reduction in NRP-1 expression by qPCR and immunoblotting analysis at different time points (Supplementary Figure 1C, 1D). Moreover, NRP-1 knockdown significantly affected the growth kinetics of BxPC-3 cancer cells *in vitro* as observed by the MTT assay for cell viability (Supplementary Figure 1E). Orthotopic pancreatic tumors were grown in athymic rats and followed till day 56 after implantation surgery. Ultrasound-targeted microbubble destruction (UTMD) of shNRP-1 minicircle was performed for NRP-1 silencing at day 28 post-implantation in a subset of animals. Total RNA and protein were extracted from the tumor tissues. qPCR data (Figure 4A) and immunoblotting data (Figure 4B) demonstrate successful silencing of NRP-1 in shNRP-1 minicircle delivered nude rats. Tumor tissues obtained from gene delivered animals were stained with H&E to characterize tissue type and morphology (Figure 4C) and extent of fibrosis by Masson's trichrome staining. We observed a typical ductal morphology and increased collagen content, features consistent with the characteristics of PDAC. Histological assessments further revealed reduced NRP-1 expression and reduced extent of fibrosis in shNRP-1 minicircle delivered animals (Figure 4C). Investigating the role of EndMT in tumor fibrosis, our qPCR analysis demonstrated significant changes in the EndMT markers at transcript level (Figure 4A) and protein level (Figure 4B) in shNRP-1 minicircle delivered nude rats when compared to scramble minicircle delivered rats. These data were further confirmed by immunohistochemistry for NRP-1 and EndMT markers (Figure 4C). At day 56, we observed significant reductions in tumor volumes in shNRP-1 delivered animals compared to scramble treated group, as measured by volumetric assessment of explanted tumors (Figure 4D). Representative CEU perfusion images of orthotopic pancreatic tumors on day 28 (before gene delivery) and day 56 (28 days after gene delivery) are shown in Figure 4E. CEU assessments at day 28 providing a pre-delivery baseline assessment, demonstrated no significant differences in normalized microvascular blood volume (MBV) and microvascular blood flow (MBF) between different tumors (Figure 4G, 4H). For perfusion imaging, the blood pool signal measured from a region of interest placed in the left ventricular (LV) cavity of the heart was used to normalize tumor plateau acoustic signal for systemic microbubble concentration [69]. Tumor area (Figure 4F) and perfusion (Figure 4G, 4H) was found to be greater and more diffuse throughout the tumor in scramble minicircle delivered rats, while reduced perfusion was observed in tumors treated with shNRP-1 minicircle. At day 56, the plateau signal intensity from tumors, representing MBV, was markedly reduced in the group treated with shNRP-1 minicircle as compared with scramble minicircle (Figure 4G). Moreover, tumor MBF was moderately reduced in the group treated with shNRP-1 minicircle as compared with scramble minicircle (Figure 4H). Given the transfection efficacy of ultrasound-mediated gene delivery coupled with delivery of high *in vivo* transfection efficiency of minicircle vectors, we would expect the shRNA knockdown to last up to 4–6 weeks in our animals. Day 56 in our study is 28 days after gene delivery, thus our significant knockdown of NRP-1 at the mRNA and protein level as consistent with prior studies using minicircle DNA vectors [70, 71]. We cannot exclude the possibility that NRP-1 expression would have been re-gained by certain cell populations by this time, as transfection is not equal in each and every cell type. However, we observed significant knockdown of NRP-1 till day 28 presumably due to high transfection efficiency of shRNA-minicircle and ultrasound mediated delivery.

## DISCUSSION

Pancreatic ductal adenocarcinoma remains the most malignant and common type of pancreatic cancer. Although considered rare in terms of incidence, it remains the 5^th^ cause of cancer related deaths, with the lowest five-year survival rate [1, 5]. Some of the important characteristics include; lack of early clinical symptoms and poor prognosis, multifaceted and complex genetic alterations and high incidences of distant metastases that contribute to the low median survival. Morphologically, PDAC is characterized by a dense fibrotic reaction termed as desmoplasia [14, 17, 18]. While previously considered to be benign, this unique compartment within the tumor has been shown to contribute to several events that initiate and promote carcinogenesis. In fact about 80–90% of PDAC tumor volume consists of this cancer-supportive compartment. Treating these patients becomes challenging owing to this dense stromal barrier, primarily composed of fibroblasts, making anti-fibrotic therapy a potential treatment modality.

The origin of CAFs, contributing to the formation of dense stroma, is a subject gaining importance lately. These fibroblasts are responsible for releasing potentially oncogenic signals, such as TGFβ, VEGF and other growth factors. Among several potential mechanisms for their origin, EndMT has gathered attention recently [34, 35]. Although first described during embryonic heart development, recent data suggest that maladaptive EndMT plays a major role in several fibrotic pathological disorders including pulmonary fibrosis, renal fibrosis and cancer [29–33, 39]. Loss of endothelial cell surface markers and gain of mesenchymal cell markers represent the hallmark of this phenomenon. Notably, EndMT is a multifactorial process driven by molecular mediators like TGFβ1. Therefore, investigating the role of novel biological clues and targets regulating EndMT can provide mechanistic insights into the underlying molecular processes involved in PDAC progression. More importantly, validation of such targets can lead to potential treatment options for fibrosis-related disorders, including PDAC. Here, we highlight EndMT as a potential source of CAFs in PDAC, with a discussion of proposed role of an angiogenic co-receptor, putative mechanisms and therapeutic implications.

The role of neuropilins in carcinogenesis, particularly the NRP-1 isoform, has been well documented [43]. Originally discovered for its role during embryonic nervous system development, the discovery of its function as a co-receptor for VEGFR-2 during angiogenesis [49] prompted investigators to study its function in tumor angiogenesis and carcinogenesis. Subsequently, several studies highlighted that NRP-1 was aberrantly expressed in several cancer types, including PDAC, and was involved in a plethora of cancer initiating and promoting pathways due to its distinctive ability to bind numerous growth factors and ligands [44, 45, 50–53, 60, 61]. Moreover, the ability of NRP-1 to exert co-receptor function to TGFβ1 opened new realms for investigation into oncogenic processes like EMT [51, 56–58]. Together, these NRP-1 findings in conjunction with studies highlighting the role of TGFβ signaling in EndMT, helped us postulate that NRP-1 could be playing a previously unrecognized regulatory role in TGFβ1-induced EndMT and potentially contributes towards tumor fibrosis.

Our correlation data obtained from human PDAC xenografts, in addition to *in vitro* knockdown and overexpression studies, led to an important observation in this study; NRP-1 plays a previously undetermined regulatory role in TGFβ-induced EndMT and associated fibrosis. Indeed, knockdown of NRP-1 inhibited, while overexpression of NRP-1 promoted EndMT and associated fibrosis. Our studies on human PDAC tissues in this report demonstrated a positive correlation between NRP-1 levels, EndMT markers and pro-fibrotic genes' expression (Figure 1A–1E). We observed that NRP-1 was expressed in all the tissue samples, however, with varying degrees. Moreover, the fibrosis and immunohistochemistry quantification results confirmed the significant correlation between NRP-1 levels and the extent of fibrosis (Figure 1A, 1B). These promising human data prompted us to specifically focus further on the role of NRP-1 in EndMT-induced tumor fibrosis. To our knowledge, this is the first study to report a novel function of NRP-1 in modulating TGFβ1-induced EndMT and associated tumor fibrosis.

By measuring the NRP-1 transcript and protein expression along with the assessment of its *in vitro* angiogenic role, we confirmed success of our NRP-1 knockdown (Figure 2A–2D) and overexpression (Figure 3A–3D) studies in HUVECs. Furthermore, we observed that loss of endothelial NRP-1 repressed EndMT characteristics. TGFβ1-mediated distinct morphological changes from cobblestone-like endothelial cell morphology to an enlarged spindle shaped, fibroblast like morphology (Figure 2E) and cytoskeletal protein organizational changes were inhibited upon NRP-1 silencing (Figure 2F). These microscopic modifications were in complete accordance with the changes in transcript (Figure 2G) and protein (Figure 2H, 2I) expression of endothelial (VE-cadherin and CD31) and mesenchymal (N-cadherin and αSMA) cell markers. Several evidences have highlighted the role of TGFβ1 signaling as a primary mediator in EndMT [36, 37, 39]. Therefore, we assessed this signaling pathway following NRP-1 silencing and overexpression. HUVEC-specific loss of NRP-1 down-regulates and deactivates TGFβ signaling, culminating in reduced phosphorylated SMAD-mediated Slug, CTGF, and Collagen 1 transcription (Figure 2H, 2K). Reduction in Slug expression in turn up-regulates the expression of the endothelial markers VE-cadherin and CD31, which inhibits the EndMT process. Furthermore, decreased levels of NRP-1 led to reduced TGFβ signaling and associated expression of TGFβ-responsive pro-fibrotic genes (Figure 2H, 2J, 2K). As anticipated, the overexpression studies demonstrated contrasting results when compared to the knockdown studies. NRP-1 overexpression promoted EndMT associated phenotypic switching in addition to the corresponding changes in expression of cell surface markers (Figure 3E–3I). Additionally, increased levels of NRP-1 led to an elevated TGFβ signaling and associated expression of TGFβ-responsive pro-fibrotic genes (Figure 3H, 3J, 3K). In contrast to TGFβ1 stimulation combined with NRP-1 overexpression, NRP-1 when overexpressed in the absence of TGFβ1 was unable to facilitate the complete transitioning of endothelial cell into mesenchymal cell. The reason behind this phenomenon could be that the regulatory role of NRP-1 in EndMT is not independent of TGFβ1. Thus our knockdown and overexpression studies provide, for the first time, solid evidence of the involvement of NRP-1 in regulating TGFβ1-induced EndMT as a potential source of CAFs. Recently, in a study of sprouting angiogenesis Aspalter *et al.* reported, using loss- and gain-of-function experiments that NRP-1 negatively regulated SMAD2/3 activation downstream of TGFβ1 and Bmp9 signaling [72]. In another study, Hirota *et al.* reported that NRP-1 and β8 integrin cooperatively balanced TGFβ signaling in brain endothelial cells. Particularly, shNRP-1 produced enhanced pSMAD3 levels upon TGFβ stimulation [73]. These studies contrast with our current findings showing that NRP-1 enhances canonical TGFβ signaling in the process of EndMT, and suggest that the function of NRP-1 differs between EndMT and sprouting angiogenesis. The response of endothelial cells to TGFβ is extremely complex and can occur through two antagonistic pathways: ALK5/SMAD2,3 or ALK1/SMAD1,5. In the case of TGFβ's failure to induce canonical SMAD2/3 signaling, TGFβ may interact with ALK1 (type I receptor) and activate SMAD1 and SMAD5. In endothelial cells, this leads to an increase in cell proliferation and migration [74]. Additionally, in the absence of canonical signaling, non-SMAD (non-canonical) pathways are also activated by TGFβ through either phosphorylation or direct interaction of its receptors. These non-SMAD pathways include various branches of MAP kinase (MAPK) pathways, Rho-like GTPase signaling pathways, and phosphatidylinositol-3-kinase (PI3K)/AKT pathways [75]. Although the activation of these other pathways cannot be ruled out, our observations regarding reduced TGFβ ligand levels upon NRP-1 silencing prompted us to mainly focus on the EndMT relevant canonical SMAD2/3 pathway. Furthermore, neuropilins can also promote non-canonical TGFβ signaling [76] and the activation of these non-canonical pathways can suppress canonical signaling [43]. These pathway interactions might be of importance in the angiogenesis studies outlined above. We hypothesize that the endothelial cell response in the NRP-1/TGFβ interaction is highly context dependent, and these cells respond differently under different physiological/pathological conditions.

**Figure 5:**
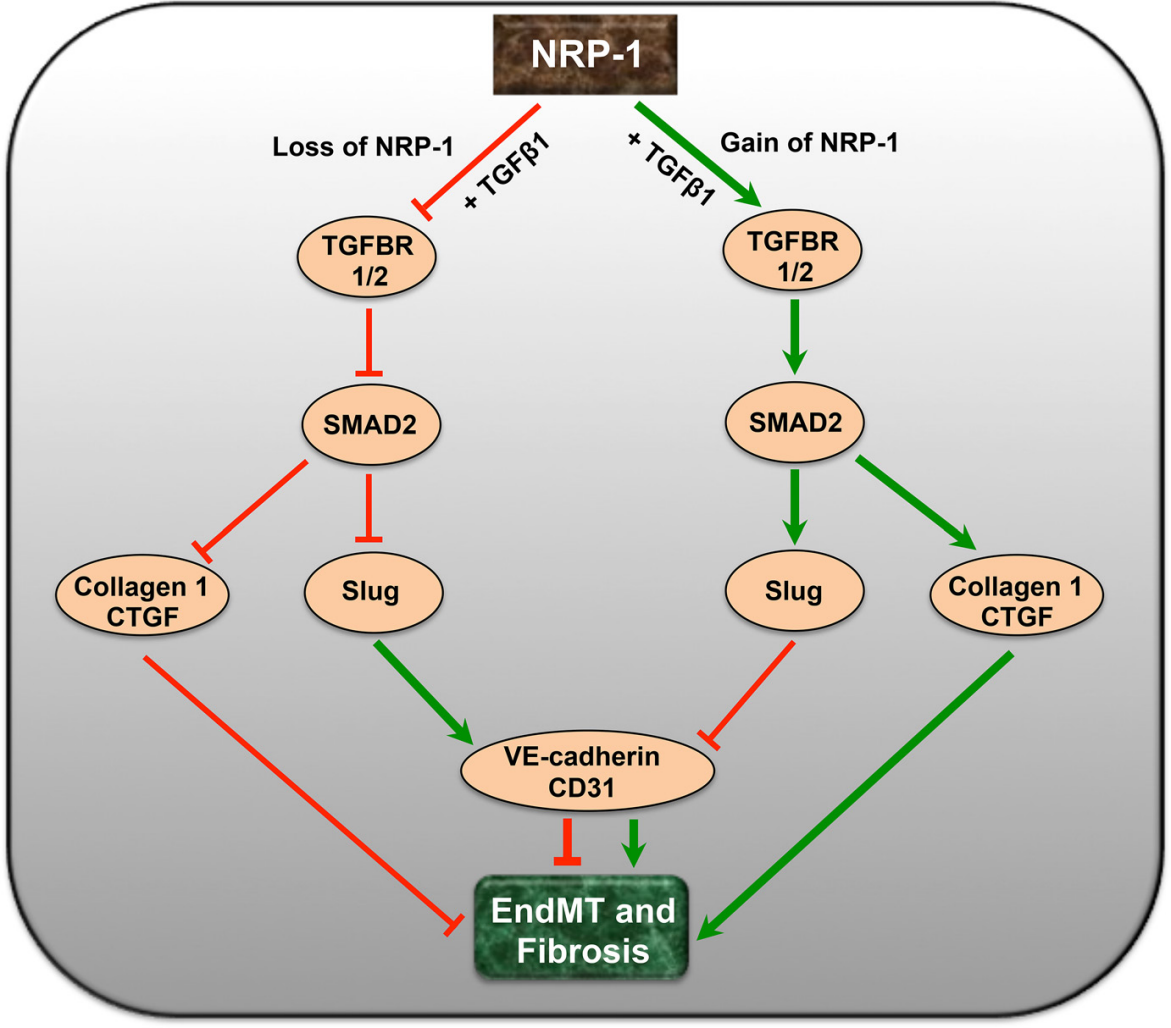
A putative schematic diagram depicting the complex interaction of NRP-1 and TGFβ-signaling pathway in EndMT and tumor fibrosis Endothelial cell-specific loss of NRP-1 down-regulates and deactivates TGFβ signaling and its receptors leading to reduced pSMAD2, Slug, CTGF and Collagen 1. The reduction in the levels of transcription factor Slug further up-regulates the expression of endothelial markers like VE-cadherin and CD31, limiting EndMT. Contrastingly, overexpression of NRP-1 exacerbates the TGFβ-induced EndMT and associated tumor fibrosis.

To directly link the preliminary *in vitro* knockdown studies with the pathobiology of tumor fibrosis, we further tested the effects of NRP-1 silencing in a clinically relevant orthotopic model of PDAC in athymic rats. This cell line can also be directly injected into the pancreas to establish orthotopic tumors [77–79]. However, we opted for the surgical implantation technique to establish our orthotopic tumors, as implanted tumors closely mimic the clinical course of the disease observed in human pancreatic cancer patients. We also preferred the implantation technique because direct injection of cells into the pancreas is sometimes associated with possible leakage, failure of primary tumor establishment and inconsistent metastatic patterns. The rats were delivered with shRNA-minicircle DNA vector targeted against NRP-1 using UTMD 28 days after tumor implantation, a validated non-invasive technique for gene delivery [80–82]. In our study we observed that at day 28 post-implantation, the tumor attained a size that was easily detectable by contrast enhanced ultrasound. We recorded baseline tumor perfusion at day 28 that was subsequently used for normalization to the perfusion recorded at day 56. This was important in our study as normalization ruled out the size/perfusion variations observed between different animals at the time of gene delivery and gave us an accurate estimate of size and perfusion reductions after gene delivery. Twenty-eight days after shNRP-1 minicircle delivery, rats demonstrated modest but significant NRP-1 knockdown along with basal increase in the endothelial markers, down-regulation of mesenchymal markers and TGFβ1-responsive Slug and CTGF expression (Figure 4A, 4B, 4C) and reduced tumor fibrosis (Figure 4C). Furthermore, the shNRP-1 delivered animals demonstrated a modest reduction in tumor volume, tumor area, microvascular blood volume and blood flow, compared to the scramble group (Figure 4D–4H). These *in vivo* results suggest that loss of NRP-1 could potentially lead to reduced tumor growth, angiogenesis and fibrosis possibly through limiting EndMT as well as reducing NRP-1-VEGFR-2 signaling. We did not examine longer time points for longevity or survival advantage of NRP-1 knockdown. Survival analysis by Kaplan-Meier estimates will be a focus of our future studies along this line of investigation. A putative mechanistic schematic representation is shown to summarize the effect of endothelial cell loss and gain of NRP-1 on EndMT and tumor fibrosis (Figure 5).

Our *in vitro* and *in vivo* studies have further validated that TGFβ1-induced EndMT can be modulated in response to manipulations of NRP-1 levels. In our experiments, we also observed a critical role of the transcription factor Slug that exacerbates the process of EndMT as previously published [39]. The exact role of other signaling pathways and transcription factors is not entirely clear at this point and warrants further mechanistic work. The recent discoveries of EndMT in various pathologies, including cancer, suggest that modulating EndMT may represent a promising novel treatment option. Studies have demonstrated that NRP-1 knockdown increases chemosensitivity towards chemotherapeutic drugs in a variety of cancer cells, including pancreatic cancer cells [61, 83]. Of interest, recently Zheng et al. highlighted the role of EMT in drug resistance and the potential of combining EMT inhibition with chemotherapy for the treatment of pancreatic cancer [84]. However, the exact contribution of EndMT towards drug resistance and the mechanistic role of NRP-1 in EndMT-dependent chemosensitivity are unknown. Moreover, the therapeutic benefit of inhibiting NRP-1-mediated EndMT combined with chemotherapy necessitates further investigation.

In conclusion, we provide herein for the first time, solid evidence of a novel anti-NRP-1 therapy that could lead to a reduction in pancreatic tumor fibrosis and growth via a regulatory effect on TGFβ1-induced EndMT. We believe that therapies directed at targets like NRP-1 to inhibit EndMT have tremendous translational implications as they could delay PDAC tumor progression, possibly owing to impaired angiogenesis as well as limited CAF recruitment.

## MATERIALS AND METHODS

### Cell culture and RNA interference

Human umbilical vein endothelial cells (HUVECs, Lonza) were grown in endothelial cell growth medium-2 (EGM™-2 Bulletkit™; Lonza) containing growth factors, serum and antibiotics. SiRNA-mediated NRP-1 knockdown studies were performed with 5 nM siNRP-1 or scramble control (Ambion) and the Dharmafect-4 transfection reagent (Dharmacon) following the manufacturer's guidelines. Recombinant human TGFβ1 (R&D Systems) was used for the TGFβ1-induced EndMT assays at a concentration of 10 ng/ml.

### Lentivirus-mediated NRP-1 overexpression studies

HUVECs were transduced with lentiNRP-1 or Empty (referred to as lentiControl) vectors (Applied Biological Materials) according to manufacturer's protocols and cells were cultured as described above.

### Quantitative real time – polymerase chain reaction (qPCR)

HUVECs were cultured with either siNRP-1, lentiviral-NRP-1 or respective controls and at 24, 48 and 72 h post-transfection/transduction, total RNA was extracted from HUVECs using TRIzol^®^ reagent (Invitrogen). Complementary DNA (cDNA) was synthesized using iScript cDNA Synthesis Kit (Bio-Rad) and qPCR was performed using SYBR Green (Quanta Biosciences) following manufacturer's protocols. Gene expression assays were performed with forward and reverse primers for *NRP-1, CD31, VE-cadherin, αSMA (α-Smooth Muscle Actin)*, *N-cadherin, Slug, TGFβ1, TGFBR1, TGFBR2, Collagen 1, CTGF (Connective Tissue Growth Factor)* and *GAPDH* (Supplementary Table S1). Data analysis was performed using manufacturer's integrated web-based software package (StepOne software v2.1) with cycle threshold (Ct)-based fold-change calculations.

### Immunoblotting and immunostaining

Transfected/transduced HUVECs were harvested and cell lysates were prepared in RIPA buffer (Santa Cruz). Equal amounts of protein were loaded on SDS polyacrylamide gel (Invitrogen) and processed for immunoblotting analysis as previously described [13, 34]. The protein expression levels of NRP-1 (Santa Cruz Biotechnology # sc-5541), CD31 (Cell Signaling # 3528), VE-cadherin (Santa Cruz # sc-6458), N-cadherin (Abcam # ab76057), αSMA (Abcam # ab5694), α-actinin (Cell Signaling # 3134), TGFβ1 (Abcam # ab9758), TGFBR1 (Cell Signaling # 3712), TGFBR2 (Cell Signaling #11888), SMAD2 (Cell Signaling # 3122), pSMAD2 (Cell Signaling # 3101), CTGF (Abcam # ab6992), Slug (Cell Signaling # 9585) and GAPDH (Santa Cruz # sc-25778) were measured using specific antibodies. GAPDH was used as a loading control for all immunoblotting experiments. Following incubation with the appropriate horseradish peroxidase-tagged secondary antibodies (Bio-Rad), signals were visualized with an enhanced chemiluminescence detection system for immunoblotting (SuperSignal^TM^, Life Technologies). Following 24 h of transfection and 48 h of transduction, immunofluorescence and immunohistochemistry for NRP-1, CD31, VE- cadherin, αSMA, N-cadherin and α-actinin were performed and visualized using standard immunostaining protocols. Fluorescent microscopy Images were captured using the Zeiss LSM700 confocal microscope and ZEN imaging software was used for image processing.

### Capillary-like tube formation assay

60 μl of Matrigel^TM^ (Becton Dickinson) was added to each well of the 96-well tissue culture plate (Corning) and allowed to polymerize at 37°C, 5% CO_2_ for 45 minutes under sterile conditions. 0.5 × 10^4^ transfected/transduced HUVECs were plated onto the surface of the Matrigel^TM^ and cultured as described earlier. All of the wells were stimulated with VEGF (50 ng/ml) to induce *in vitro* tube formation. The capillary-like network formation was observed at regular intervals until 24 h and photomicrographs were recorded at different time intervals for quantification.

### Cell viability assay

MTT assay of cell viability (Vybrant, Life technologies) was performed to assess growth kinetics according to the manufacturer's protocol. 10,000 BxPC-3 cells transfected with shRNA or scramble minicircle were seeded in each well of 96 well plates (*n* = 6). 10 μl of the 12 mM MTT stock solution was added to each well and incubated at 37°C, 5% CO_2_ for 4 hours. 50 μl of DMSO (Sigma) was added to each well and mixed thoroughly to solubilize the formazan. The pate was again incubated at 37°C, 5% CO_2_ for 10 minutes. Each sample was mixed again and absorbance was read at 540 nm using an automated plate reader (Molecular Devices).

### Human xenograft studies

Formalin-fixed paraffin-embedded and flash frozen human PDAC tissues were obtained from Dr. David Hedley (OICR, Toronto, Canada). qPCR and immunoblotting for NRP-1, EndMT and fibrosis markers was performed on these samples. Immunohistochemistry, hematoxylin-eosin (H&E) staining and Masson's trichrome staining (Polybiosciences Inc.) was performed on the human PDAC tissue following the manufacturer's guidelines. NRP-1 levels and fibrosis were quantified with light microscopy using the ImageJ software (NIH).

### Generation of orthotopic tumor model in athymic rats

The *in vivo* study protocol was approved by the Animal Care and Use Committee at the Keenan Research Centre for Biomedical Science, Li Ka Shing Knowledge Institute, St Michael's Hospital, University of Toronto. Rowett Nude (RNU, strain 316) rats were purchased from Charles River. BxPC-3 human pancreatic cancer cells were purchased from ATCC and cultured in RPMI 1640 medium (ATCC) supplemented with 10% fetal bovine serum and antibiotics. (5 × 10^6^) cells were injected (200 μl cells in RPMI medium + 200 μl Matrigel^TM^) in the flank of athymic rats to establish heterotopic tumors. Pancreatic tumors grown subcutaneously were then harvested at exponential growth phase under aseptic conditions to establish the orthotopic model. Viable tissues were cut and minced into 3-mm^3^ pieces. In recipient RNU rats, the tail of the pancreas, located in the splenorenal ligament within the spleen, was gently exteriorized via laparotomy. Five tumor fragments were sutured with 7–0 surgical sutures onto the pancreas so that tumor tissue is completely surrounded by pancreatic parenchyma. The pancreas was placed back in the original position and the muscle and skin were sutured. Post-mortem, tumor tissues were stained with H&E staining and Masson's trichrome staining for characterization.

### Contrast enhanced ultrasound perfusion imaging and ultrasound-mediated loss of NRP-1 *in vivo*

The contrast-enhanced ultrasound (CEU) perfusion and gene delivery studies were performed as previously described [80, 81]. CEU perfusion imaging of tumors was performed at 4 and 8 weeks after implantation surgery. At 4 weeks post tumor implantation surgery, ultrasound-targeted microbubble destruction (UTMD) [80–82] was performed for NRP-1 silencing. For UTMD, shNRP-1 minicircle (SBI Biosciences) DNA vector (200 μg) was charge coupled to cationic microbubbles (1 × 10^9^) and the suspension was administered intravenously during the external application of high power ultrasound over the pancreas (*n* = 4). Scramble minicircle (SBI Biosciences) DNA vector (200 μg) was delivered by UTMD as an appropriate control in a separate group of animals (*n* = 4). Animals were sacrificed 4 weeks after gene delivery (8 weeks post implantation). Tumor tissue and remote organs were collected for evaluation of tumor volume and other downstream analyses as previously described.

### Statistical analysis

All data were expressed as mean ± SD unless otherwise indicated. The Student's *t*-test was applied when the means of two groups were being compared. Differences between multiple means were evaluated by One-way ANOVA (GraphPad Prism5) followed by Tukey's post-hoc test to compare individual means. *P* value of < 0.05 was considered to be statistically significant. Spearman's Rho test was applied to perform bivariate correlation analysis between two variables, where values of R closer to 1 indicated positive correlation.

## SUPPLEMENTARY MATERIALS


