# Supplementary material for: Novel regulatory role of neuropilin-1 in endothelial-to-mesenchymal transition and fibrosis in pancreatic ductal adenocarcinoma
Source: Oncotarget. 2016 Aug 11;7(43):69489–506. doi: 10.18632/oncotarget.11060 (PMC5342493; doi:10.18632/oncotarget.11060)
Supplement: Supplementary file 1 [file oncotarget-07-69489-s001.pdf]

Novel regulatory role of neuropilin-1 in endothelial-to-mesenchymal transition and fibrosis in pancreatic ductal adenocarcinoma

Supplementary Materials

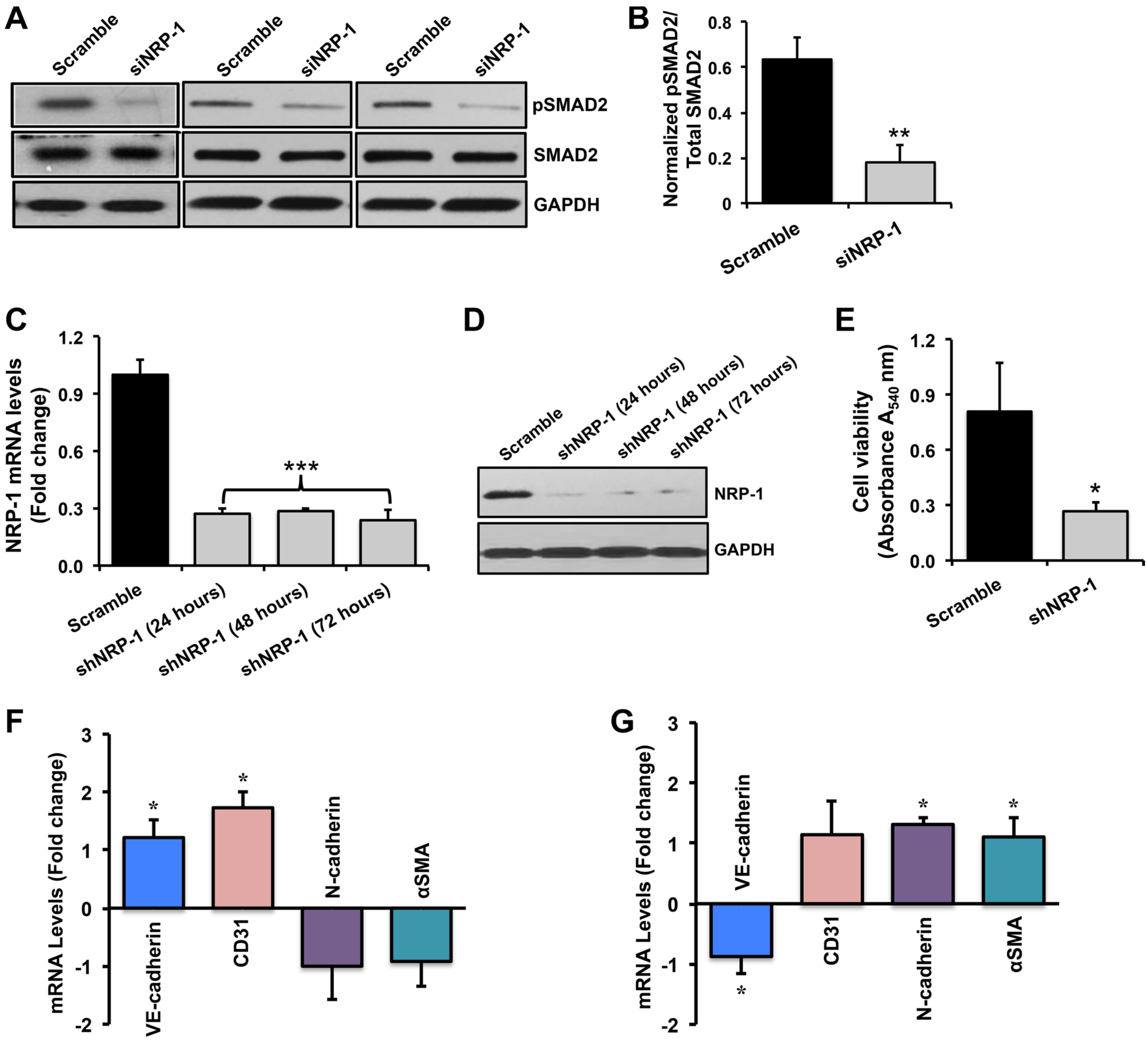

Supplementary Figure S1: Regulatory role of NRP-1 in TGFβ1-induced EndMT is dependent on TGFβ1. (**A, B**) Immunoblotting data analysis demonstrates significant down-regulation of SMAD2 phosphorylation (pSMAD2) at protein level upon NRP-1 silencing. $p < 0.01$ vs. scramble control. $n = 3$ in triplicate. (**C, D**) BxPC-3 cells were cultured and transfected with 5 µg of either shNRP-1 or scramble control minicircle using electroporation technique. Total RNA and protein was extracted from the transfected cells at 24, 48 and 72 h post-transfection. (C) qPCR data demonstrate successful silencing of NRP-1 in siNRP-1 transfected HUVECs after 24 h. All qPCR data are presented as fold change to the scrambled control. $***p < 0.001$ vs. scramble control. $n = 3$ in triplicate. (D) NRP-1 silencing was confirmed at protein level by immunoblotting at 24, 48 and 72 h after transfection. GAPDH was used as a loading control. (**E**) Loss of NRP-1 significant affected the growth kinetics of BxPC-3 cells when tested for viability using MTT assay. $*p < 0.05$ vs. scramble control. $n = 6$. (**F**) Loss of NRP-1 in the absence of TGFβ1 significantly increased the expression of endothelial markers; VE-cadherin and CD31, however, the decrease in expression of mesenchymal markers; αSMA and N-cadherin, although moderate, was not significant as assessed by qPCR. $*p < 0.05$ vs. scramble control. $n = 3–4$ in triplicate. (**G**) Furthermore, NRP-1 overexpression in the absence of TGFβ1 stimulation resulted in an increase in mesenchymal markers' expression but no significant decrease in the expression of endothelial cell marker CD31 assessed in the study at transcript level. $*p < 0.05$ vs. lentiControl $n = 3–4$ in triplicate.

Supplementary Table S1: List of primers for qPCR

Genes	Nucleotide Sequences (Primers)
Hs-NRP-1-Forward	5′-CCCAACAGCCTTGAATGCAC-3′
Hs-NRP-1-Reverse	5′-ATTTCTAGCCGGTCGTAGCG-3′
Hs-CD31- Forward	5′-CCTTCTGCTCTGTTCAAGCC-3′
Hs-CD31- Reverse	5′-GGGTCAGGTTCTTCCCATTT-3′
Hs-VE-cadherin- Forward	5′-ACAGAGCTCCACTCACGCTC-3′
Hs-VE-cadherin- Reverse	5′-CATGAGCCTCTGCATCTTCC-3′
Hs-Slug- Forward	5′-TCGGACCCACACATTACCTT-3′
Hs-Slug- Reverse	5′-GCAGTGAGGGCAAGAAAAAG-3′
Hs-N-cadherin- Forward	5′-GTGCATGAAGGACAGCCTCT-3′
Hs-N-cadherin- Reverse	5′-CCACCTTAAAATCTGCAGGC-3′
Hs-CTGF-Forward	5′-CAGGCTAGAGAAGCAGAGCC-3′
Hs-CTGF-Reverse	5′-TGGAGATTTTGGGAGTACGG-3′
Hs-TGFBR1-Forward	5′-GCTGCTCCTCCTCGTGCT-3′
Hs-TGFBR1-Reverse	5′-TTGTCTTTTGTACAGAGGTGGC-3′
Hs-TGFBR2-Forward	5′-CTGCACATCGTCCTGTGG-3′
Hs-TGFBR2-Reverse	5′-GGAAACTTGACTGCACCGTT-3
Hs-TGFβ1-Forward	5′-CCCTGGACACCAACTATTGC-3′
Hs-TGFβ1-Reverse	5′-CTTCCAGCCGAGGTCCTT-3
Hs-Collagen 1A1-Forward	5′-AAGAGGAAGGCCAAGTCGAG-3′
Hs-Collagen 1A1-Reverse	5′-CACACGTCTCGGTCATGGTA-3′

List of the nucleotide sequences of the primers used to amplify genes for qPCR experiments. (Hs; Homo sapiens).
